# Supplementary material for: Aerosols overtake greenhouse gases causing a warmer climate and more weather extremes toward carbon neutrality
Source: Nat Commun. 2023 Nov 9;14:7257. doi: 10.1038/s41467-023-42891-2 (PMC10636203; doi:10.1038/s41467-023-42891-2)
Supplement: Supplementary file 1 — Supplementary Information [file 41467_2023_42891_MOESM1_ESM.pdf]

Supporting Information for

Aerosols overtake greenhouse gases causing a warmer climate and more weather extremes toward carbon neutrality

Pinya Wang[1], Yang Yang[1*], Daokai Xue[2], Lili Ren[3], Jianping Tang[2], L. Ruby Leung[4], Hong Liao[1]

[1]Jiangsu Key Laboratory of Atmospheric Environment Monitoring and Pollution Control, Jiangsu Collaborative Innovation Center of Atmospheric Environment and Equipment Technology, Joint International Research Laboratory of Climate and Environment Change, School of Environmental Science and Engineering, Nanjing University of Information Science and Technology, Nanjing, Jiangsu, China

[2]School of Atmospheric Sciences, Nanjing University, Nanjing, Jiangsu, China

[3]College of Environment and Ecology, Jiangsu Open University, Nanjing, Jiangsu, China

[4]Atmospheric Sciences and Global Change Division, Pacific Northwest National Laboratory, Richland, Washington, USA

Correspondence to: Y. Yang, yang.yang@nuist.edu.cn

Contents of this file

Table S1 to S6

Figures S1 to S14

Table S1 Global mean concentrations of major greenhouse gases in 2020, 2050 and 2100 following the carbon neutrality scenario.

GHGs	2020	2050	2100
CO_2	414.04 ppm	437.48 ppm	399.51 ppm
CH_4	1884.13 ppb	1429.12 ppb	1060.96 ppb
N_2O	331.88 ppb	343.54 ppb	352.96 ppb
CFC-11	218.24 ppt	138.92 ppt	61.86 ppt
CFC-12	495.08 ppt	366.11 ppt	231.71 ppt

Table S2 Information for the 13 CMIP6 GCMs that provide global surface air temperature and precipitation for 2050 under SSP1-1.9.

Model	Institution/Country	Resolution (LatxLon)
CAMS-CSM1-0	CAMS/China	1.125°x1.125°
CanESM5	CCCma/Canada	2.8°x2.8°
CNRM-ESM2-1	LASG-IAP/China	1.4°x1.4°
EC-Earth3-Veg-LR	EC-Earth-Consortium/Europe	1.4°x1.4°
EC-Earth3-Veg	EC-Earth-Consortium/Europe	0.7°x0.7°
EC-Earth3	EC-Earth-Consortium/Europe	0.7°x0.7°
GFDL-ESM4	NOAA-GFDL/USA	1°x1.25°
GISS-E2-1-G	NASA-GISS/USA	2.5°x2°
IPSL-CM6A-LR	IPSL/France	1.26°x2.5°
MIROC-ES2L	MIROC/Japan	2.8°x2.8°
MIROC6	MIROC/Japan	1.4°x1.4°
MRI-ESM2-0	MRI/Japan	1.125°x1.125°
UKESM1-0-LL	MOHC/UK	1.25°x1.875°

Table S3 Simulation design to attribute the future climate change under the carbon neutrality to the effects of individual changes in GHGs, aerosols, and tropospheric O_3.

Simulation	GHGs	Aerosols	Tropospheric O_3
Baseline	2020	2020	2020
GHG2050	2050	2020	2020
AerGHG2050	2050	2050	2020
All2050	2050	2050	2050
All2100	2100	2100	2100

Table S4 Information for the 6 selected CMIP6 GCMs that provide global vertical profiles of O_3.

Model	Institution/Country	Resolution (Lat × Lon)
CNRM-ESM2-1	CNRM/France	1.4° × 1.4°
FGOALS-g3	LASG-IAP/China	2.25°× 2°
GFDL-ESM4	NOAA-GFDL/USA	1°× 1.25°
IPSL-CM6A-LR	IPSL/France	1.26°× 2.5°
MRI-ESM2-0	MRI/Japan	1.125° × 1.125°
UKESM1-0-LL	MOHC/UK	1.25°x1.875°

Table S5 Information for the CMIP6 GCMs that provide monthly global surface air temperature from SSP245, SSP245-GHG and SSP245-Aer in Detection and Attribution Model Intercomparison Project (DAMIP). Available models are ticked for each experiment.

Model	Institution/Country	Resolution	SSP24.5	SSP245-GHG	SSP245-Aer
AWI-CM-1-1-MR	AWI/ Germany	0.9375°x 0.9375°	√		
BCC-CSM2-MR	BCC/ China	1.125°x1.125°	√		
CAMS-CSM1-0	CAMS/China	1.125°x1.125°	√		
CanESM5	CCCma/Canada	2.8°x2.8°	√	√	√
CESM2	NCAR/USA	0.9°x1.25°	√		
CESM2-WACCM	NCAR/USA	0.9°x1.25°	√		
CIESM	THU/China	1.25°x0.9375°	√		
CMCC-CM2-SR5	CMCC/Italy	1.25°x0.9375°	√		
EC-Earth3	EC-Earth-Consortium/Europe	0.7°x0.7°	√		
EC-Earth3-Veg	EC-Earth-Consortium/Europe	0.7°x0.7°	√		
FGOALS-f3-L	CAS/China	1°x1°	√		
FGOALS-g3	CAS/China	2.25°x2°	√		
GFDL-CM4	NOAA-GFDL/USA	1°x1.25°	√		
GFDL-ESM4	NOAA-GFDL/USA	1°x1.25°	√		
GISS-E2-1-G	NASA-GISS/USA	2.5°x2°	√	√	√
HadGEM3-GC31-LL	MOHC NERC/UK	1.25°x1.875°	√	√	
INM-CM4-8	INM/Russia	2.5°x1.5°	√		
INM-CM5-0	INM/Russia	2.5°x1.5°	√		
IPSL-CM6A-LR	IPSL/France	1.26°x2.5°	√	√	
KACE-1-0-G	NIMS-KMA/Korea	1.875°x 1.25°	√		
KIOST-ESM	KIOST/Korea	1.875° x 1.5°	√		
MIROC6	MIROC/Japan	1.4°x1.4°	√	√	√
NorESM2-LM	NCC/Norway	1.875°x2.5°	√	√	√
NorESM2-MM	NCC/Norway	0.9375°x1.25°	√		

Table S6. Definitions of five climate extreme indices used in this work.

Extremes	Definitions
Heat waves	Daily Tmax exceeds its threshold and lasts for no less than 3 days. The temperature threshold is defined as the annual maximum Tmax of the baseline period (2020 in this study).
Humid heat waves	Similar to above, but derived from daily maximum wet-bulb temperatures (TW).
R10mm	Number of heavy precipitation days. A heavy precipitation event is defined as the daily precipitation (RR) exceeding 10 mm d^{-1}.
CWD	Maximum number of consecutive days with RR>= 1mm
Pretotal	Total precipitation on wet days RR>= 1mm

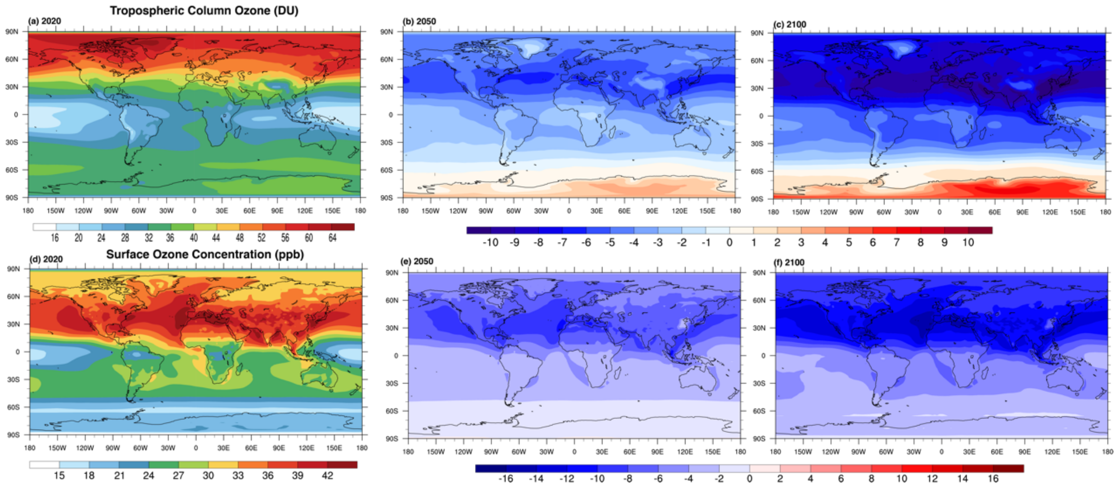

Figure S1 The spatial patterns of 2020 tropospheric column O_3, TCO (a, unit: DU) and surface O_3 (d, unit: ppb), and changes in TCO (b&c) and surface O_3 (e&f) in 2050 and 2100.

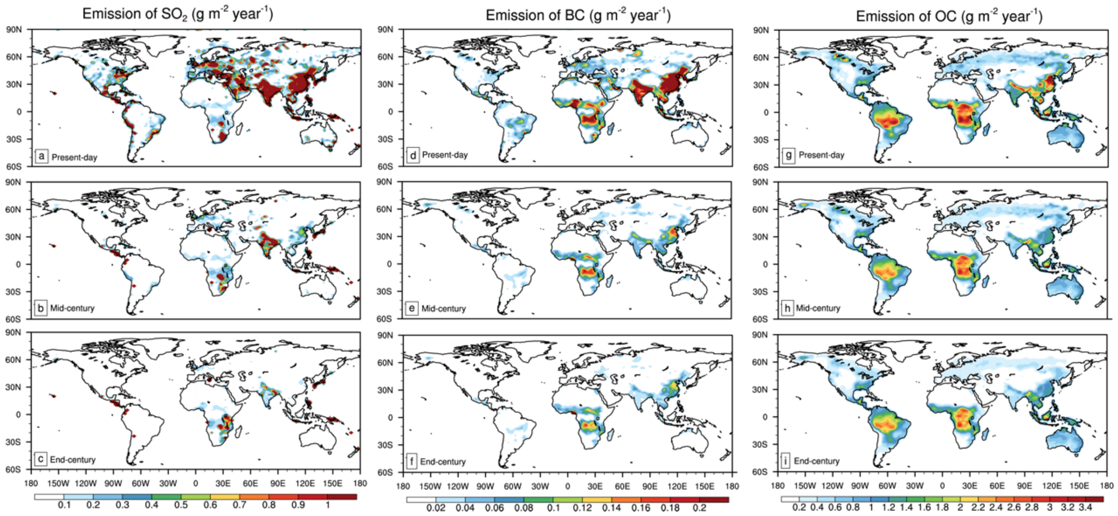

Figure S2 The annual mean emissions of SO_2 (a, b&c), BC (d, e&f) and OC (g, h&i) in 2020 (top), 2050 (middle) and 2100 (bottom). Unit: g m^{-2} a^{-1}.

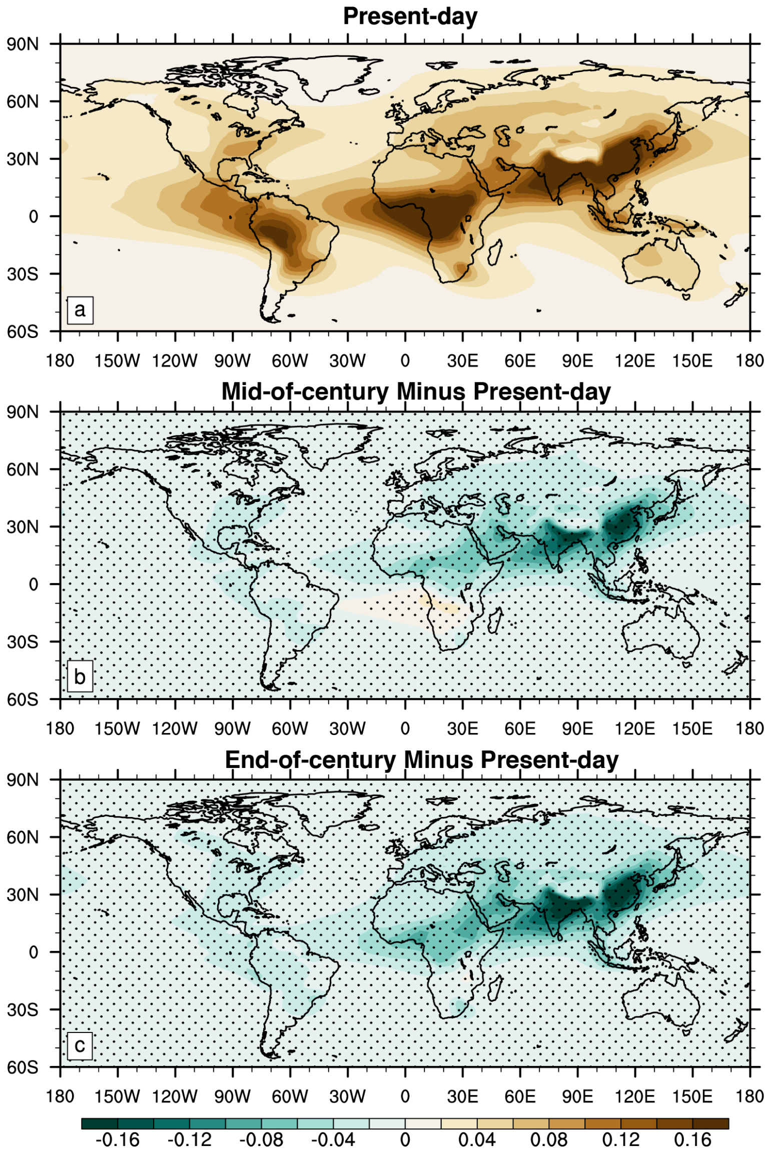

Figure S3 The spatial distributions of aerosol optical depth (AOD) (at 500 nm) in 2020 (a), and its changes in 2050 (b) and 2100 (c), relative to the 2020 levels. The stippled areas indicate statistical significance with 95% confidence from a two-tailed Student's t test.

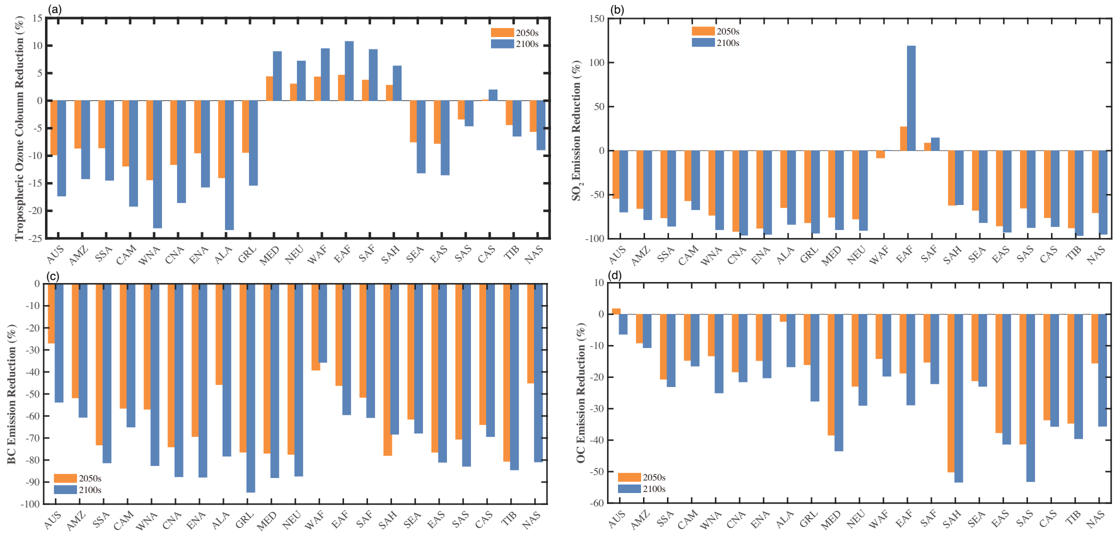

Figure S4 Regional mean reductions in TCO (a) and emissions of SO_2 (b), BC (c) and OC (d) in 2050 and 2100, relative 2020. Unit: %. The locations of the 21 subregions here are outlined in Figure S5.

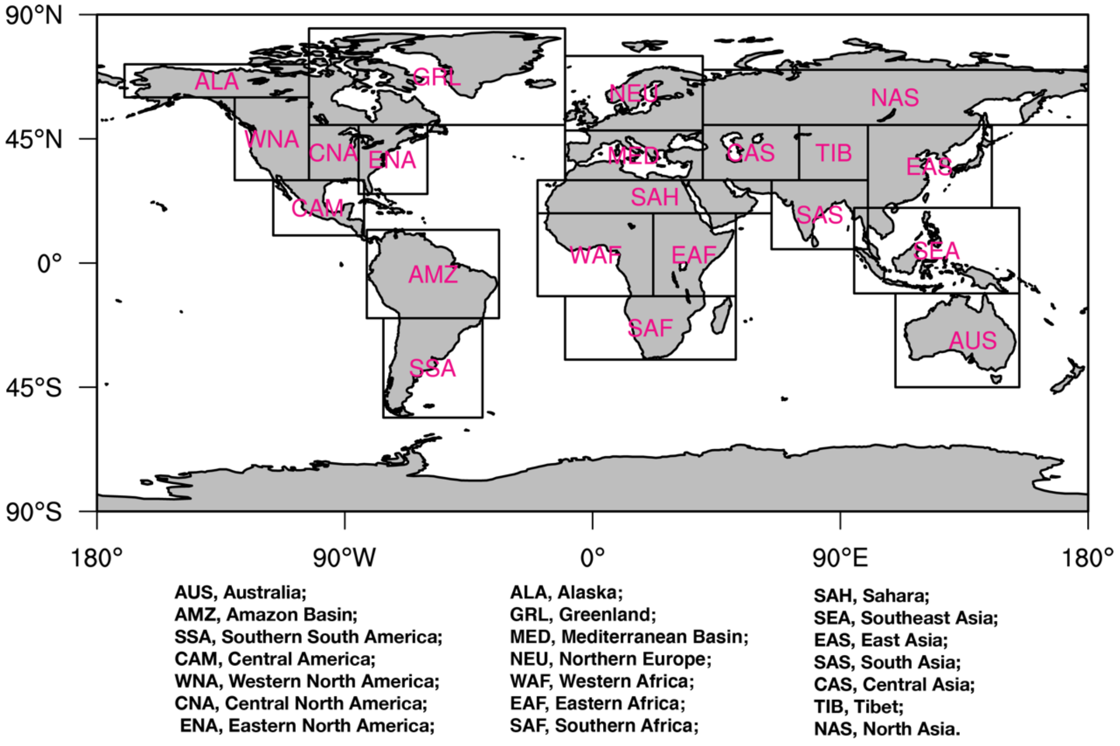

Figure S5 Outlines of 21 subregions for regional attribution analysis.

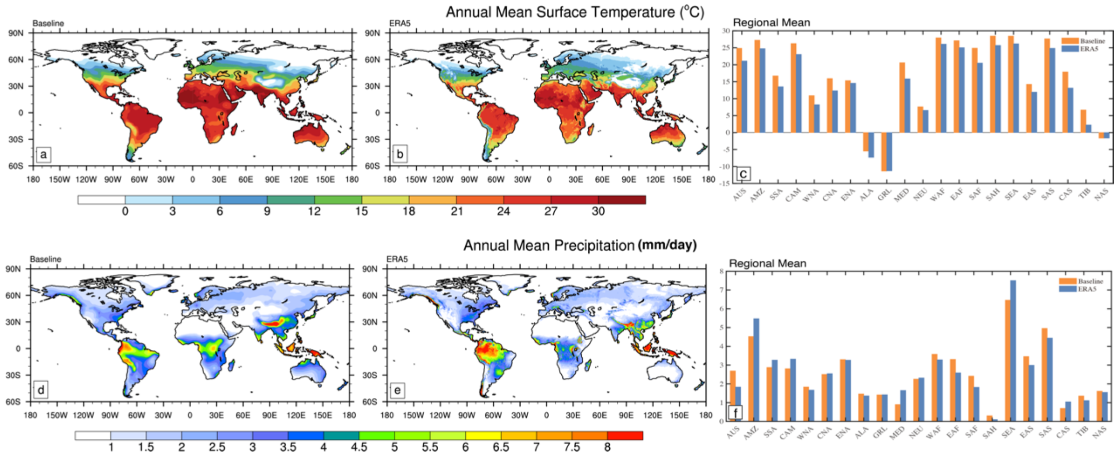

Figure S6 The annual mean surface air temperature (a&b) and precipitations (d&e) in the Baseline simulation and ERA5 reanalysis for 2006-2010. Regional mean surface temperature and precipitation are presented in panels c&f.

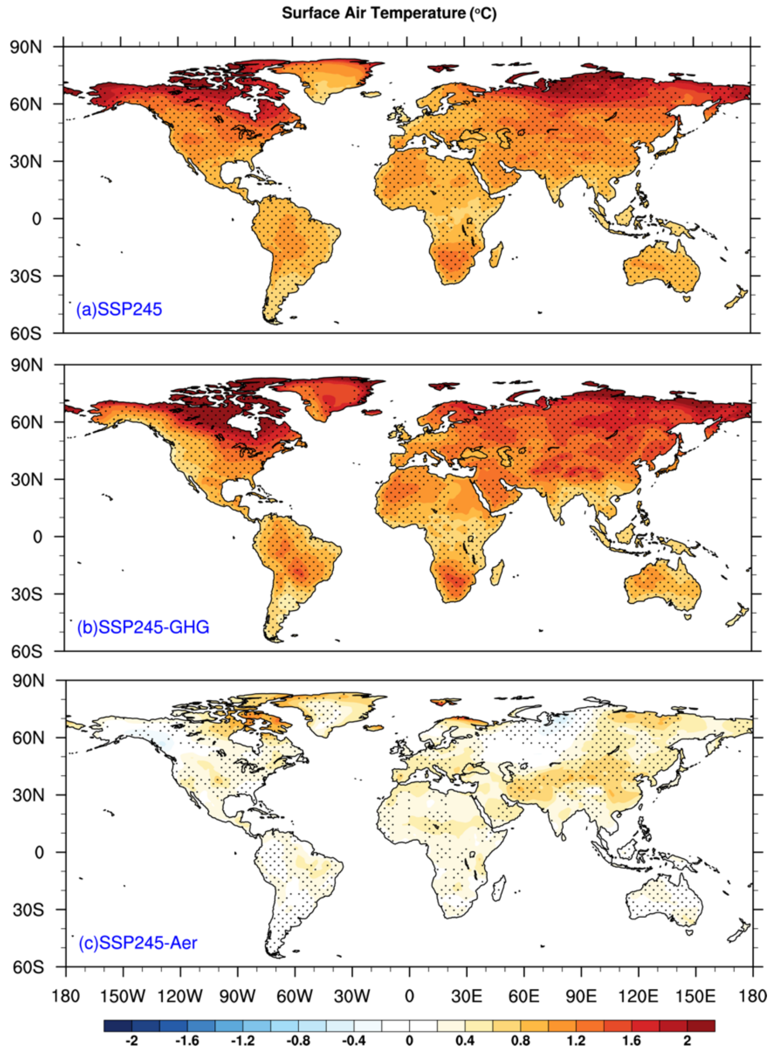

Figure S7 Changes in annual mean surface temperature (°C) of the CMIP6 multi-model ensemble mean in 2050 under SSP245 (a), SSP245-GHG (b) and SSP245-Aer (c) relative to the 2020 levels. Stippling indicates more than half of the simulations show consistent changes.

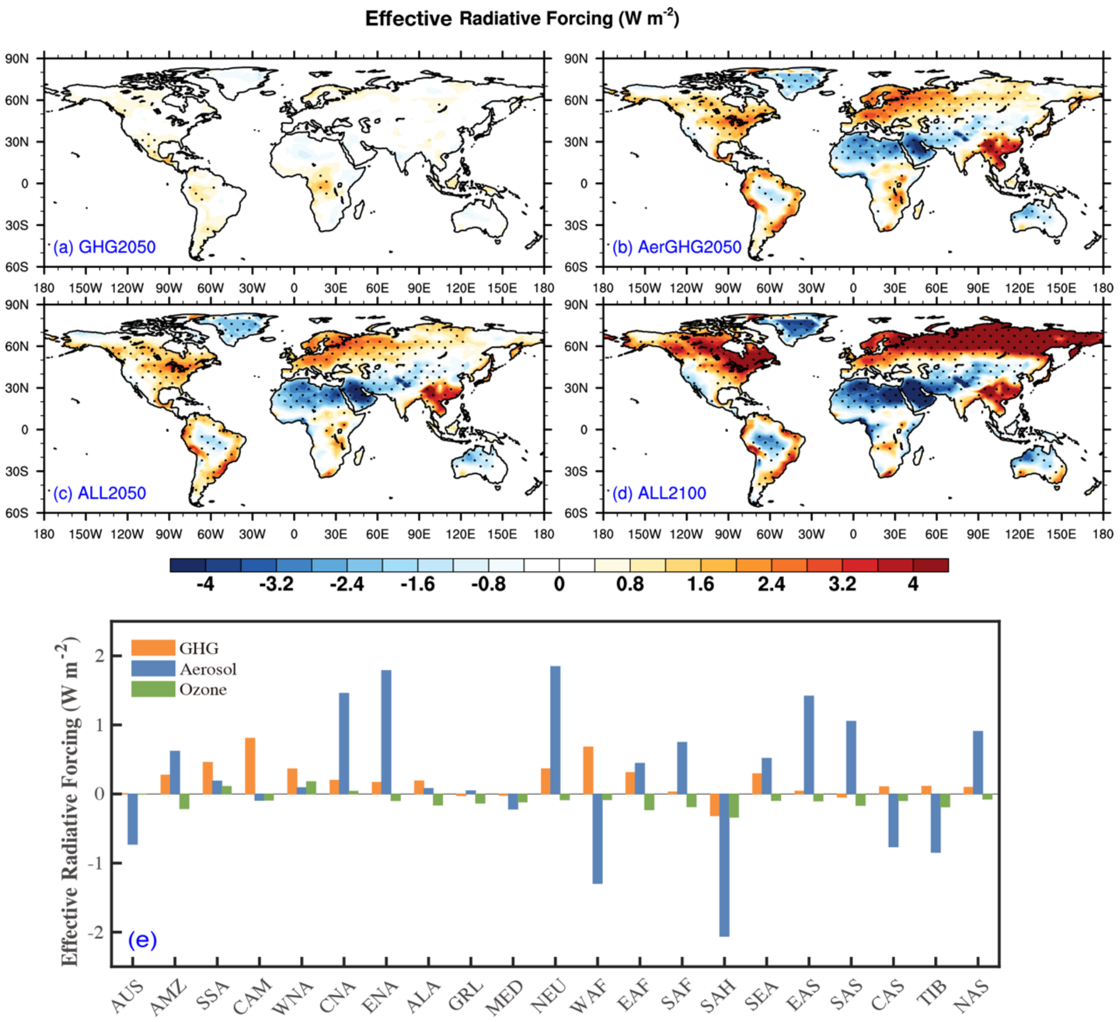

Figure S8 Changes in effective radiative forcing (ERF) at top of atmosphere $(W/m2)$ in GHG2050 (a), AerGHG2050(b), ALL2050 (c), and ALL2100 (d), relative to Baseline (2020). Regional mean changes in ERF at top of atmosphere in 2050 attributed to GHGs, aerosols and tropospheric O_3 changes over 21 subregions (e). The stippled areas indicate statistical significance with 95% confidence from a two-tailed Student's t-test.

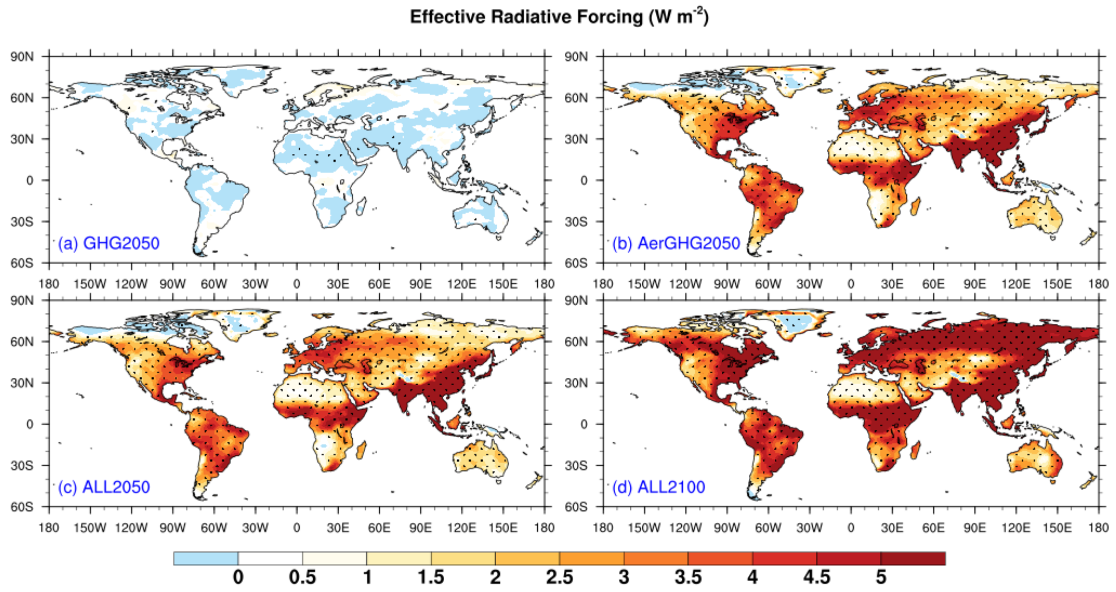

Figure S9 Similar to Fig. S8 but for changes in effective radiative forcing (ERF) at surface (W/m^2).

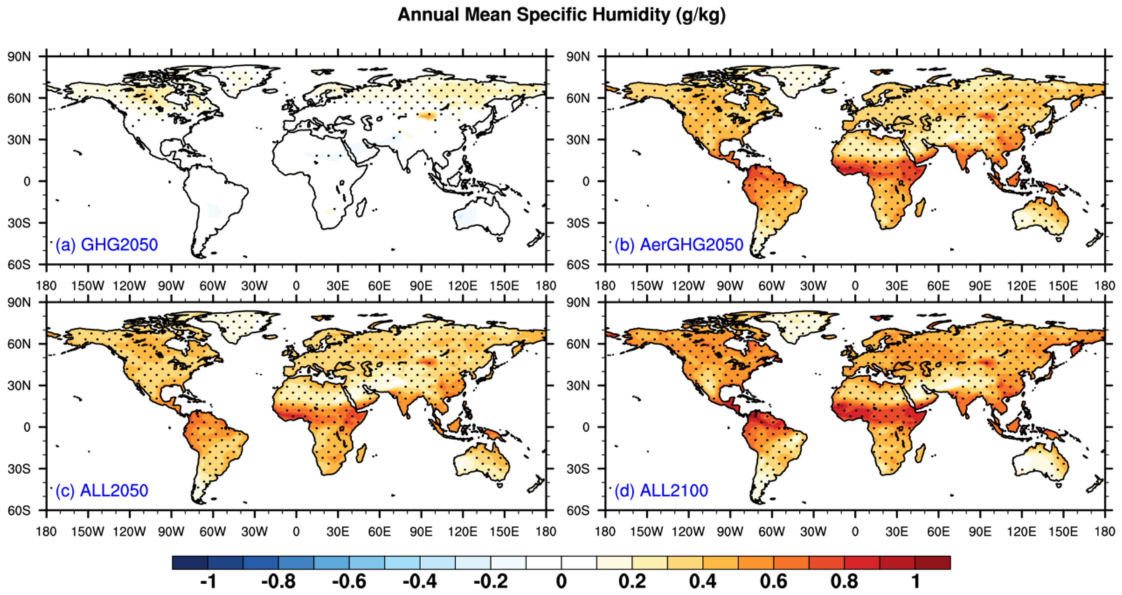

Figure S10 Changes in specific humidity (g/kg) in GHG2050 (a), AerGHG2050(b), ALL2050 (c), and ALL2100 (d), relative to Baseline (2020). The stippled areas indicate statistical significance with 95% confidence from a two-tailed Student's t-test.

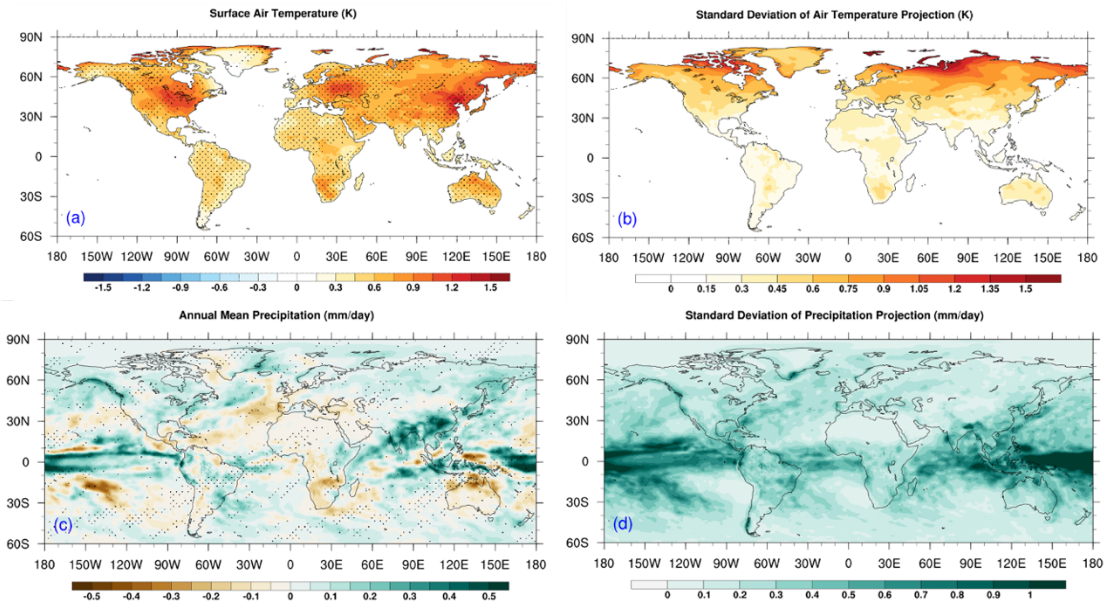

Figure S11 (a)&(c) Changes in annual mean surface air temperature (°C) and precipitation (mm/day, b) of the CMIP6 multi-model ensemble mean in 2050 under SSP1-1.9, relative to the 2020 levels. Stippling indicates more than half of the simulations show consistent changes. (b)&(d) Standard deviation of changes in surface air temperature and precipitation for the 13 CMIP6 models.

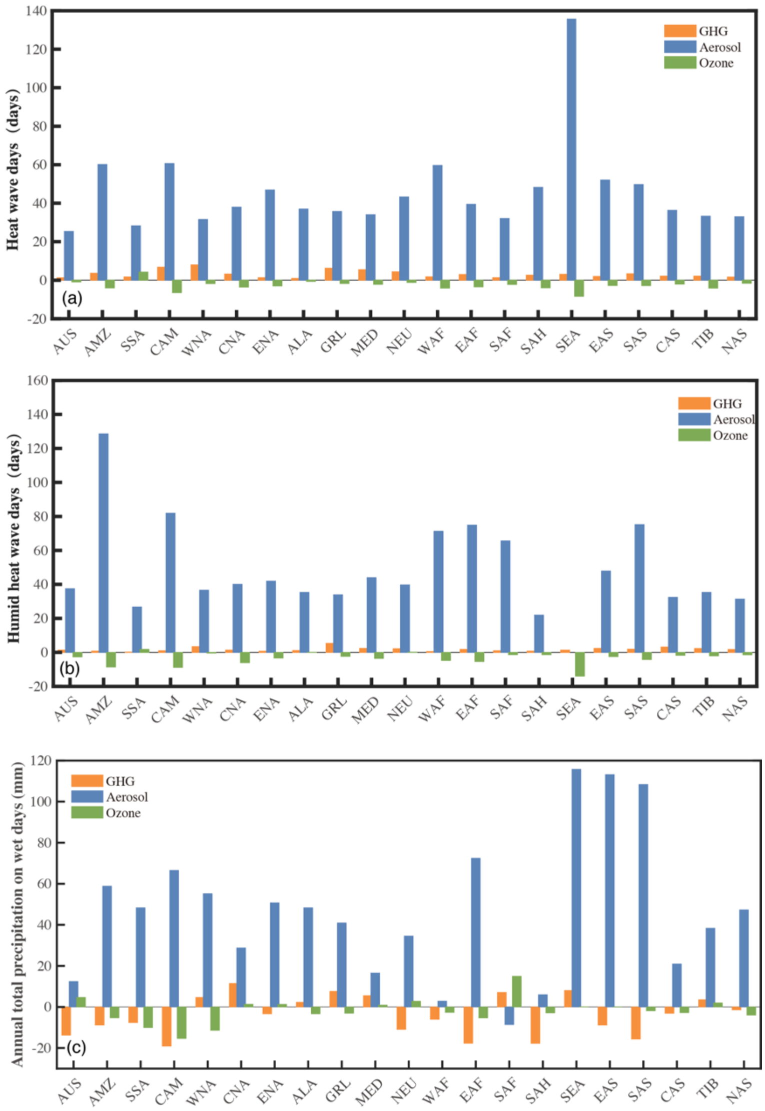

Figure S12 The regional mean changes in heat wave frequency (e, unit: days), humid heat wave frequency (b, unit: days) and Pretotal (c, unit: mm) in 2050 attributed to GHGs, aerosols and tropospheric O_3 changes over 21 subregions.

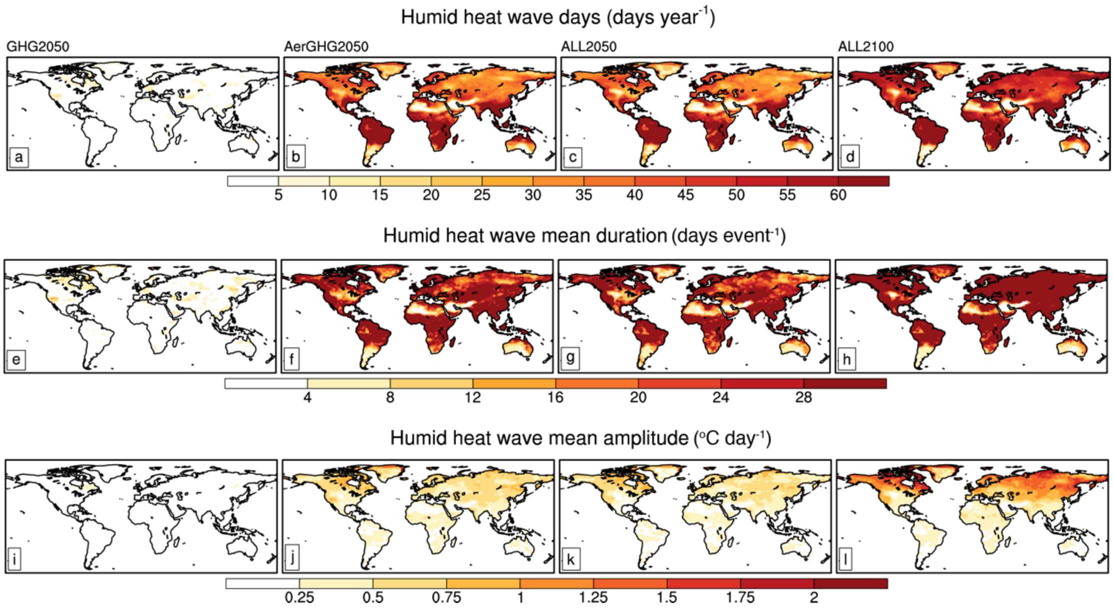

Figure S13 Humid heat wave days, mean duration and amplitude in GHG2050, AerGHG2050, ALL2050 and ALL2100.

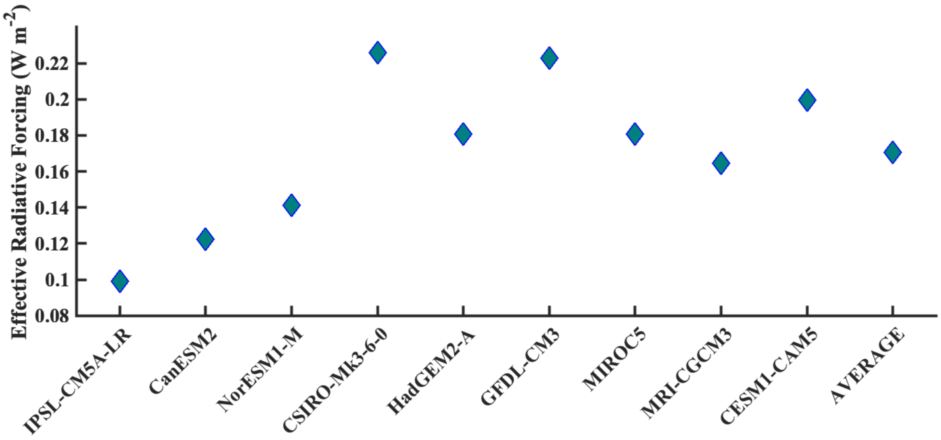

Figure S14 Estimated global average effective radiative forcing (ERF, W/m^2) at the top of the atmosphere due to aerosol changes in 2050 relative to 2020 levels. ERF values in other global climate models other than CESM1 are calculated by multiplying ERF in CESM1 by the ratios of historical ERF (2000 Vs. 1860) between the other GCMs derived from ref. 51 and CESM1.
